# Advances in Chitosan-Based Smart Hydrogels for Colorectal Cancer Treatment

**DOI:** 10.3390/ph17101260

**Published:** 2024-09-25

**Authors:** Urszula Piotrowska, Klaudia Orzechowska

**Affiliations:** Department of Pharmaceutical Chemistry and Biomaterials, Faculty of Pharmacy, Medical University of Warsaw, 1 Banacha Str., 02-097 Warsaw, Poland

**Keywords:** chitosan, natural polymers, hydrogels, stimuli-responsive hydrogels, drug delivery systems, colorectal cancer, anticancer therapy

## Abstract

Despite advancements in early detection and treatment in developed countries, colorectal cancer (CRC) remains the third most common malignancy and the second-leading cause of cancer-related deaths worldwide. Conventional chemotherapy, a key option for CRC treatment, has several drawbacks, including poor selectivity and the development of multiple drug resistance, which often lead to severe side effects. In recent years, the use of polysaccharides as drug delivery systems (DDSs) to enhance drug efficacy has gained significant attention. Among these polysaccharides, chitosan (CS), a linear, mucoadhesive polymer, has shown promise in cancer treatment. This review summarizes current research on the potential applications of CS-based hydrogels as DDSs for CRC treatment, with a particular focus on smart hydrogels. These smart CS-based hydrogel systems are categorized into two main types: stimuli-responsive injectable hydrogels that undergo sol-gel transitions in situ, and single-, dual-, and multi-stimuli-responsive CS-based hydrogels capable of releasing drugs in response to various triggers. The review also discusses the structural characteristics of CS, the methods for preparing CS-based hydrogels, and recent scientific advances in smart CS-based hydrogels for CRC treatment.

## 1. Introduction

According to the American Cancer Society, there will be over 2 million new cancer cases and over 611 thousand cancer deaths in the US in 2024. Improved treatment and earlier detection for some cancers have led to a continued decline in cancer mortality through 2021, averting over 4 million deaths since 1991 [1].

Colorectal cancer (CRC) is the third most common malignancy worldwide, accounting for approximately 10% of all cancer cases. Despite improvements in early detection and treatment in developed countries, the lifetime risk of CRC development is around 5%. The 5-year survival rate for CRC is less than 10%, making it the second-leading cause of cancer-related deaths globally [2]. CRC-related mortality is a result of the progressive accumulation of multiple genetic and epigenetic aberrations within cells [3]. CRC usually emerges from the glandular, epithelial cells of the large intestine. Normally, new epithelial cells are replenished every 2–7 days to replace those continually shed by the passage of food, which makes intestinal epithelium the most vigorously self-renewing tissue of adult mammals [4]. Any dysregulation occurring from proliferation to differentiation stages, and even post-differentiation, may lead to uncontrolled cell division and the formation of tumors. Various factors can contribute to this dysregulation, including specific hereditary gene mutations (such as hereditary non-polyposis colorectal cancer and fibroblast activation protein) and mutagenic and carcinogenic factors (high-fat diets, excessive alcohol consumption) [5]. The human large intestine is one of the most densely populated microbial communities on Earth, so the impact of infectious factor is also very important. Studies in animal models revealed the role of several bacteria in carcinogenesis (*Helicobacter pylori*, *Streptococcus bovis*, *Fusobacterium nucleatum*, certain strains of *Escherichia coli*, and producing enterotoxins *Bacteroides fragilis* strains) [6,7]. What is more, colon cancer has been divided into left-sided colon cancer, which is the more common type of cancer, and right-sided colon cancer, found in the colonic spleen [8]. The differences in the gut microbiota on both the left and right side of the colon are important factors in determining the occurrence and prognosis of CRC [9]. Moreover, the treatment of the right- and left-sided colon cancer are also different [10].

The principal modes of cancer therapy are surgery, radiotherapy, and chemotherapy, either alone or in combination [11]. Currently, chemotherapy with cytostatic drugs remains the most common method for cancer treatment [12]. Chemotherapeutic drugs induce apoptosis of rapidly growing cancer cells by interfering with DNA synthesis and mitosis in a non-selective manner, while causing undesirable side effects in normal tissues that reduce patient survival [13].

An interesting approach to the targeted delivery of chemotherapeutic agents into tumors is drug delivery systems (DDSs) [14,15]. DDSs could improve the effectiveness of the therapy by controlled release, while simultaneously protecting active substances from enzymatic degradation, enhancing drug accumulation in tumor tissues, meeting immunogenicity requirements, and reducing the systemic side effects of the therapy.

Of all the approaches, hydrogels have played a significant role as a carrier in drug 66 delivery, as they are biodegradable and biocompatible materials [16]. The range of their applications has been expanding to include a variety of drug and dosage forms. In the case of CRC, hydrogels facilitate localized treatment due to their adhesive properties or ability to gel in situ in response to temperature changes, reducing systemic toxicity and improving patient outcomes. They can be designed to release drugs in response to specific stimuli, such as pH changes in the gastrointestinal tract, making them highly adaptable for colon-targeted therapies in precision oncology. Hydrogels consist of cross-linked polymer chains forming a three-dimensional network structure, which allows them to absorb substantial amounts of fluid. Due to their high water content, soft texture, and porosity, they closely mimic the characteristics of living tissues [17]. Hydrogels can be synthesized from natural or synthetic polymers. The use of natural polymers such as polysaccharides (chitosan (CS), alginate, dextran, and cellulose) has many advantages associated with their biocompatibility and biological properties [18]. These polymers can be converted into 3D hydrogel structures through physical or chemical cross-linking approaches and become promising biomaterials for tissue engineering, wound repair, and controlled delivery of the drugs. Among the above-mentioned polymers, CS, natural, polysaccharide-based polymer has been widely exploited for the delivery of drugs, peptides, protein, and genes to the colon [19].

In this paper, state-of-the-art CS-based colon-targeted hydrogel drug delivery systems that have potential applications in the treatment of CRC were reviewed.

## 2. Physicochemical and Biological Properties of Chitosan

CS is a linear, semi-crystalline polymer obtained by the partial deacetylation of chitin [20]. This process, which can be achieved through enzymatic or chemical methods [21,22], was first discovered by Rouget in 1859 [23]. Crustacean shells are a well-established industrial source for the production of commercially available CS. Recently, however, there has been growing interest in CS extracted from agricultural waste products, such as fungal sources. This shift is largely driven by the demand for vegan-friendly products. Moreover, fungal CS has a low polydispersity index and is biocompatible due to the absence of allergenic animal-derived proteins [24,25]. CS available on the market exists in the form of dry flakes that range in color from white to yellow, solution, and fine powder [26].

**Figure 1:**
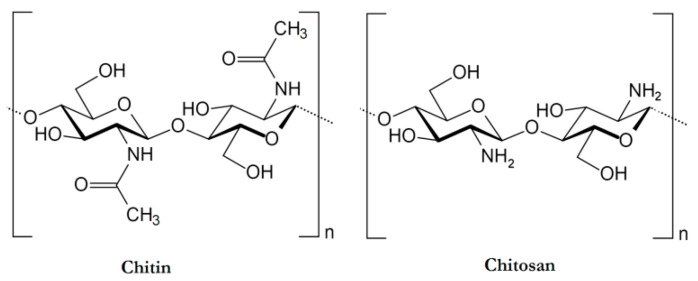
Chemical structure of chitin and chitosan. This figure is adapted from [22] with permission under CC BY 4.0 license.

The chemical structure of CS is composed of two subunits: D-glucosamine and *N*-acetyl-D-glucosamine, interconnected by β-1,4-glycosidic bonds. As a result, CS is chemically known as poly(*N*-acetyl-2-amino-2-deoxy-D-glucopyranose) (Figure 1) [27].

Each deacetylated subunit of CS contains a primary amine group with a pKa value of approximately 6.5. The cationic charge of these amino groups is responsible for CS’s mucoadhesive effect with the negatively charged mucosal surfaces of the intestinal tract. This property enables CS-based DDSs to enhance the absorption of chemotherapeutic agents, prolong their circulation, and improve therapeutic efficacy. Additionally, protonated CS transiently opens the tight junctions among epithelial cells, increasing cancer cell permeability, which makes it an excellent carrier for the local treatment of CRC [28,29]. CS can also be metabolized in vivo by enzymes such as lysozyme through enzymatic reactions [30].

CS can be characterized by its molecular weight, typically ranging from 50 kDa to 2000 kDa [31], and its degree of deacetylation (DD), which varies from 40% to 98% [32]. These two parameters significantly influence the physicochemical and biological properties of CS and can be controlled during production. Molecular weight affects the solubility, biodegradability, and antioxidant activity of the polymer. The degree of deacetylation is directly proportional to the solubility, viscosity, crystallinity, biocompatibility, mucoadhesiveness, analgesic, antibacterial, and hemostatic activity of CS [33,34]. Among various techniques, including Fourier transform infrared spectroscopy (FTIR), nuclear magnetic resonance (NMR), and UV spectroscopy, FTIR is the simplest and primary method for evaluating the DD of CS [35]. While the ideal CS structure would have a 100% DD, achieving a high DD remains challenging, limiting the use of CS in biomedical applications.

CS is soluble in aqueous acidic solution, such as acetic acid [36], citric acid, glutamic acid [37], aspartic acid, hydrochloric acid, or lactic acid [38], at pH levels below 6.5. This solubility occurs by protonating the –NH_2_ groups in the glucosamine units to form R-NH_3_^+^ [39]. This protonation leads to repulsion between the positively charged macrochains, allowing water molecules to diffuse in and subsequently solvate the macromolecules [40]. CS with a low DD (around 40%) remains soluble up to a pH of 9.0, whereas highly deacetylated CS (≥85%) is soluble only up to a pH of 6.5 [41]. The addition of salts to the CS solution further affects its solubility. As the ionic strength increases, the solubility decreases. In solution, CS initially adopts an extended conformation because each positively charged deacetylated unit repels the neighboring glucosamine unit. However, the addition of an electrolyte reduces this repelling effect, leading the molecule to adopt a more random coil-like conformation. As a result, increasing the electrolyte concentration triggers a salting-out effect, causing CS to precipitate from the solution [32,42,43].

Increasing the DD increases the viscosity of CS. This can be explained by the fact that CS with different levels of deacetylation adopts different conformations in aqueous solution. Highly deacetylated CS has an extended conformation with a more flexible chain due to charge repulsion within the molecule. In contrast, CS with a low degree of deacetylation tends to have a rod-like or coiled shape, resulting from the lower charge density along the polymer chain [41].

Protonated CS can complex with various anionic molecules, forming nanoparticles (NPs) through polyelectrolyte interactions that lead to self-assembly. These hydrophilic CS NPs exhibit longer retention times in the bloodstream. Hu et al. demonstrated that chondroitin sulfate-chitosan NPs were effectively endocytosed by Caco-2 fibroblasts without causing significant cytotoxic effects, even at high concentrations [44]. In Caco-2 cells, a reversible opening of tight junctions (TJs) by CS led to a decrease in transepithelial electrical resistance and an increase in paracellular marker fluxes [45,46]. Some studies reported that CS or its derivatives induced reorganization of the actin cytoskeleton in Caco-2 cells, while others found no morphological changes in the actin cytoskeleton [29,47]. In Caco-2 cells, CS appears to activate a PKC-dependent signaling pathway that affects TJ integrity [48].

CS and its oligomers, chitooligosaccharides (COS), act as prebiotics by promoting the growth of beneficial bacteria in the colon while suppressing harmful, proinflammatory bacteria. This modulation of gut microbiota can improve microbial balance and contribute to CRC prevention [49]. For instance, Wu et al. demonstrated that COS could protect mice models from CRC by rebalancing bacterial and fungal populations, specifically reducing harmful bacteria like *Escherichia*–*Shigella*, *Enterococcus*, and *Turicibacter*, and increasing beneficial microbes such as *Akkermansia* and *Cladosporium* [50]. Additionally, Calinescu et al. proposed innovative CS-based DDSs for targeted probiotic colon delivery, further highlighting the therapeutic potential of CS in gut health management [51].

Finally, the functional groups in CS, such as primary amine and hydroxyl groups, act as electron donors, facilitating CS’s cross-linking with other polymers and making it well-suited for DDSs.

## 3. Preparation of Chitosan-Based Hydrogels

CS hydrogels, as natural polymer hydrogels, are formed through various physicochemical interactions that result in a cross-linked network structure. The amino groups present in the CS chains play a crucial role in the formation of these hydrogels. The properties of CS hydrogels, including mechanical strength, swelling behavior, and biodegradability, can be finely tuned by manipulating several factors. These include the synthesis method, CS concentration, temperature, pH, and the duration of cross-linking. By adjusting these parameters, it is possible to customize the hydrogel to meet specific needs in various applications [52].

There are two main methods for preparing CS-based hydrogels based on their attachment mechanisms: chemical and physical cross-linking [53,54]. Chemical cross-linking involves the formation of covalent bonds between molecules, resulting in hydrogels with excellent mechanical strength. A covalently cross-linked gel is characterized by a permanent network structure due to its irreversible chemical bonds. However, most of the cross-linkers used to date are either relatively toxic or have an unclear fate in the human body, with limited data available on their biocompatibility. In contrast, physical cross-linking involves the formation of noncovalent bonds, such as van der Waals forces, hydrogen bonds, ionic bonds, and hydrophobic interactions forming “reversible” or “physical” gels [55,56].

### 3.1. Chemical Cross-Linking

Chemically cross-linked CS-based hydrogels, often referred to as “chemical gels,” are composed of a network of covalent bonds that link the polymer chains together. These hydrogels are formed when the multiple amino and hydroxyl functional groups on the chitosan chain interact with a cross-linking agent, resulting in a stable and more rigid three-dimensional (3D) gel structure. This covalent cross-linking imparts enhanced mechanical strength and stability to the hydrogels, making them suitable for various applications where durability and rigidity are required (Table 1) [57].

CS hydrogels have been synthesized using a variety of chemical cross-linkers, including glutaraldehyde [58] or epoxy-based molecules [59]. However, the use of these synthetic cross-linking agents often results in some degree of cytotoxicity, which can negatively affect the biocompatibility of CS-based DDSs. This limitation has prompted research into safer and more biocompatible cross-linking alternatives for biomedical applications. Examples of such biocompatible cross-linkers include genipin [60,61] or citric acid [62]. Interestingly, citric acid can form both ionic and covalent bonds with CS. This dual bonding capability enhances the stability and mechanical properties of the CS hydrogel, making citric acid a versatile and biocompatible cross-linker for various biomedical applications [63].

Another method to induce chemical cross-linking is through photocross-linking. During the process, light (usually UV or visible light) activates a photoinitiator or directly excites specific functional groups within the polymer chains, leading to the formation of covalent bonds among the chains [64]. This technique, used both in vivo and in vitro, allows for the regulation of drug release from the hydrogel based on the duration of light exposure. By adjusting the light-exposure time, researchers can precisely control the release profile of the drug, making this method highly effective for targeted and controlled drug delivery applications [65,66].

**Table 1:** Characterization of chemical cross-linked chitosan-based hydrogels.

Chemical Cross-Linking	Characteristics of the Hydrogels	Ref.
Schiff base reaction	High stability Self-adapting ability pH/swelling dependence	[61,67]
Diels–Alder reaction	Injectability Self-healing High mechanical qualities	[68]
Michael addition reaction	Good mechanical characteristics Structural stability Good in vivo degradability Thermal stability	[69]
Thiol-ene click chemistry	Improved mechanical strength Surface roughness Biocompatibility pH-sensitive, CS-based hydrogels	[70]
Photopolymerization	Biocompatibility (enhance cell adherence, proliferation, and differentiation) Biodegradability Wound healing Mechanically resilient Elastic hydrogel Controlled drug delivery	[71,72,73]
Graft copolymerization	Highly elastic hydrogels Bioscaffolds	[74]

### 3.2. Physical Cross-Linking

Physically cross-linked CS hydrogels, formed through non-covalent interactions, exhibit remarkable mechanical strength, reduced toxicity, and the ability to reverse their gelation process. Typically, the sol-gel transition in CS hydrogels is triggered by changes in pH, ionic strength, and temperature. The characterization of CS hydrogels formed through different physical cross-linking mechanisms is presented in Table 2.

**Table 2:** Characterization of physically cross-linked chitosan-based hydrogels.

Physical Cross-Linking	Characteristics of the Hydrogels	Ref.
Ionic interaction	Stable network structure Enhanced mechanical strength and stability Control over pore morphology and surface properties of the hydrogel Low cytotoxicity	[75]
Hydrogen bonding interaction	Unique shapes and mechanical properties, including elasticity and the ability to bear pressure-induced deformation pH-sensitive, temperature-sensitive, and dual-responsiveness CS-based hydrogels	[76]
Hydrophobic interaction	Induce structural modifications, including changes in porosity and surface area	[77]
Electrostatic interaction	Decreased degree of swelling Increased viscoelasticity Injectable hydrogel	[78]

#### 3.2.1. Cross-Linking in Different pH

The basic preparation of a CS hydrogel with physical cross-links involves solubilizing the macromolecules in an acidic solution. However, both alkaline and acidic solvent systems are important for preparing physically cross-linked CS hydrogels [79].

**Figure 2:**
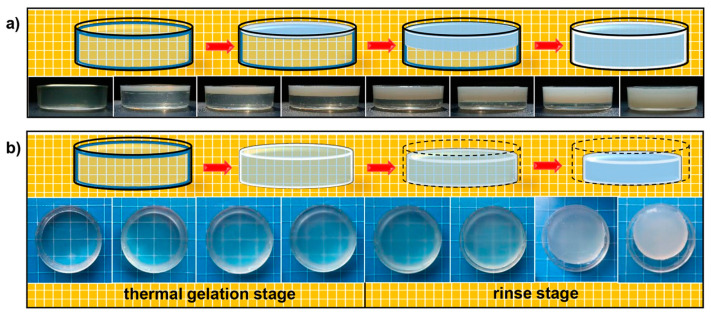
The gelation process of CS hydrogel (**a**) via acidic solvent; (**b**) via alkaline solvent. This figure is adapted from [79] with permission under CC BY 4.0 license.

In an acidic system, the gelation mechanism of CS is primarily driven by the deprotonation and entanglement of CS macromolecules, a process promoted by the diffusion of OH^−^ from the coagulation bath (Figure 2a).

In contrast, in an alkaline CS system (alkaline-urea aqueous solvent), a thermally induced gelation mechanism occurs, driven by the evolution of intermolecular hydrogen bond interactions. As the temperature rises, CS tends to form inter- and intramolecular hydrogen bonds among its own chains rather than interacting with OH^−^ ions. This leads to the aggregation of macromolecules due to their self-association tendency, with the degree of aggregation increasing as the protective effect of the solvent weakens. Consequently, the system transitions from a polymer solution to a three-dimensional network (Figure 2b) [80].

#### 3.2.2. Ionotropic Gelation of Chitosan

As mentioned, ionically cross-linked CS hydrogels offer distinct advantages over covalently cross-linked hydrogels, particularly in medical and pharmaceutical applications. Due to the biocompatibility of ionic cross-linkers, these hydrogels are generally well-tolerated by the body, making them especially promising as DDSs. The use of ionic cross-linking allows for the development of CS hydrogels that are not only effective in delivering therapeutic agents but also safer and more adaptable to various biomedical applications [55,56].

Ionotropic gelation is a simple, fast, and cost-effective process performed under mild conditions, as it does not involve the use of organic solvents [81]. In counterionic gelation, CS forms gels in the presence of counterions that neutralize its positive charge, allowing for the formation of gel networks. This process is used in pharmacy to create various drug forms, such as microcapsules, NPs, and films, which can be utilized for controlled drug release [82]. The cross-linking agents are typically charged ionic entities with well-defined molecular weights that neutralize the positive charges of CS, leading to the formation of ionic bridges between CS molecules and the subsequent creation of a gel.

**Figure 3:**
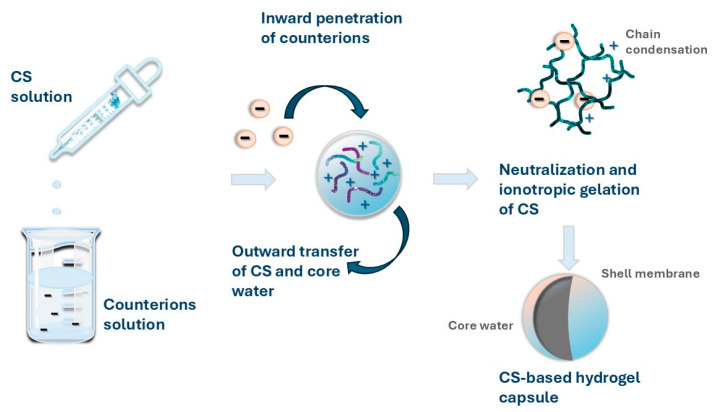
Preparation of chitosan nanoparticles by ionic gelation method.

This technique was first reported by Calvo et al. in 1997 for the preparation of CS NPs [83]. They produced CS-based hydrogel capsules by adding a CS acid solution to a solution of counterions with stirring (Figure 3).

Numerous studies have explored various counterions for CS ionotropic gelation, including tripolyphosphate [84], hexametaphosphate [85], (Fe(CN)_6_)^−4^/(Fe(CN)_6_)^−3^ [86], alginate [87], hyaluronan [88,89], chondroitin [90], κ-carragenan [91], polyaldehydrocarbonic acid [92], sulfate (such as octyl sulfate, lauryl sulfate, hexadecyl sulfate, and cetylstearyl sulfate [32]), citrate [93], and glycerophosphate [94].

## 4. Smart Chitosan-Based Hydrogels for the Treatment of Colorectal Cancer

Smart hydrogels are a new family of polymers that are capable of altering their chemical and/or physical properties upon exposure to endogenous (pH, enzymes, biomolecules, oxidation-reduction) or exogenous stimuli (e.g., temperature, light, mechanical force, magnetic or electric field, or ultrasound) [95,96,97].

**Figure 4:**
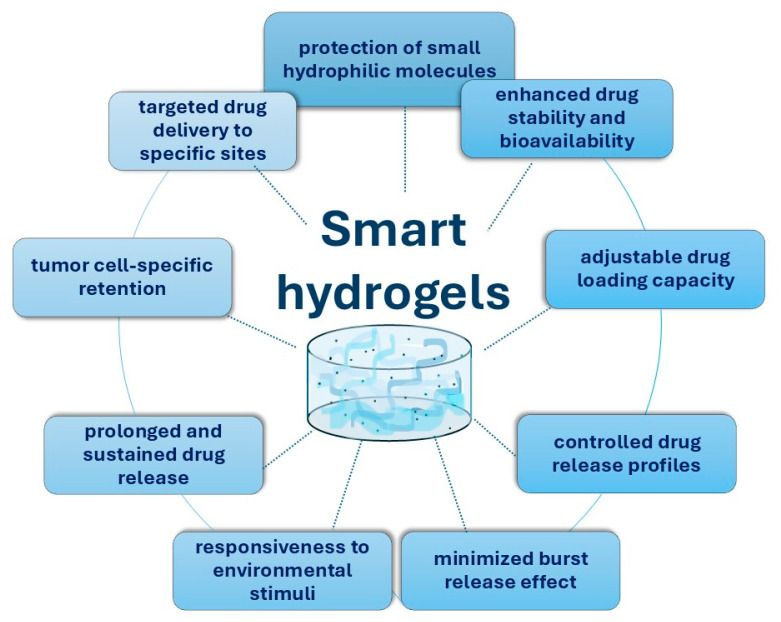
Unique properties of smart hydrogels.

Smart hydrogels possess unique properties, as shown in Figure 4. They can load and protect both small hydrophilic molecules and macromolecules, offering higher stability for prolonged circulation in the bloodstream. These hydrogels can significantly improve drug-loading efficiency and bioavailability while reducing the side effects. Being a soft material, they have a higher likelihood of specific retention at the target site, with the capacity to flatten on the vascular surface and anchor at multiple points simultaneously [97].

In this review article, smart CS-based hydrogel systems have been divided into two categories: stimuli-responsive injectable hydrogels that undergo sol/gel transition in situ, and stimuli-responsive CS-based hydrogels that are able to release drugs upon various triggers for CRC therapy.

### 4.1. In Situ-Forming Chitosan-Based Hydrogels for Colorectal Cancer Therapy

In situ gel technology has emerged as a promising approach for localized and controlled drug delivery, particularly useful in postoperative adjuvant chemotherapy for cancer patients with residual tumors following ablation surgeries [98]. This method is also gaining traction in intraperitoneal chemotherapy, which has shown potential in treating peritoneal carcinomatosis in CRC by possibly reducing mortality rates [99]. However, the challenge remains in maintaining a high intraperitoneal-to-plasma drug concentration ratio, as it is critical for ensuring that more of the drug remains in the peritoneal cavity rather than being absorbed into the systemic circulation [100].

In situ gels, which can be injected in liquid form and solidify upon exposure to specific physiological conditions or stimuli, offer unique pharmacokinetic parameters such as controlled and prolonged drug release. This technology encapsulates drugs within a polymer network in vivo with high payload efficiency, releasing them gradually through diffusion [101]. Based on the type of cross-linking trigger, in situ gels are categorized into several types, including temperature modulation, solvent exchange, UV irradiation, ionic cross-linkage, and pH change [102]. Current research is increasingly focused on developing multi-responsive gel technology for DDSs to enhance pharmacokinetics [103].

Thermosensitive hydrogels, a type of in situ gel, are particularly promising for CRC therapy. These hydrogels can be injected directly into tumor sites, where they undergo a sol-gel transition at body temperature, acting as drug reservoirs that mimic the properties of soft tissues [104]. They biodegrade naturally over time, eliminating the need for surgical removal. This method, especially when applied intraperitoneally, provides a simpler, cost-effective, and time-saving alternative to open surgery. CS-based thermosensitive, injectable hydrogels have shown great potential in CRC therapy as carriers for the sustained delivery of drugs [105]. Additionally, they may reduce peritoneal adhesion formation, which is crucial because severe adhesions can interfere with the exposure of residual tumors to the drug, thereby diminishing the effectiveness of intraperitoneal chemotherapy [106].

Despite their potential, thermosensitive CS hydrogels face significant challenges. The inherent fragility and low mechanical strength of pure CS hydrogels are due to their high water content. The numerous hydrogen bonds among CS chains create a relatively loose three-dimensional network with larger pores, which allows drug molecules to diffuse easily, leading to a burst release and limiting the hydrogel’s broader applicability in in situ DDSs [107].

Furthermore, a major challenge in developing thermosensitive CS hydrogels has been their inability to remain in solution at physiological pH [108]. When CS solutions are neutralized with strong bases, CS tends to precipitate [109]. Moreover, CS in aqueous solution does not naturally exhibit thermosensitive properties.

**Figure 5:**
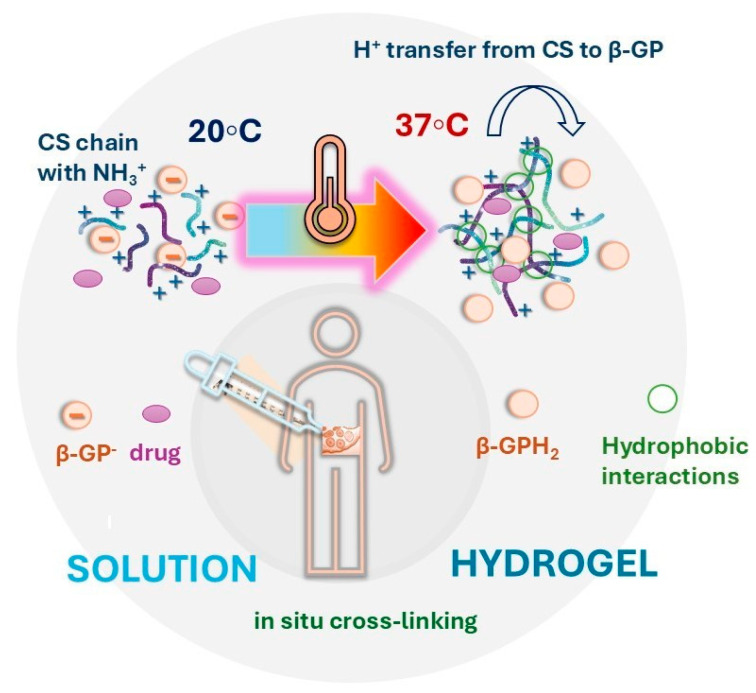
In situ gelation mechanism between chitosan and β-glycerophosphate.

The introduction of a weak base, β-glycerophosphate (β-GP), with a pKa close to that of CS (6.65 at 25 °C) [110], extends the solubility of CS to physiological pH, enabling homogeneous gelation when heating to 37 °C [111]. The gelation mechanism is attributed to the neutralization of the positively charged ammonium groups in CS, which reduces the static charge surrounding the polymer. This process is driven by the heat-induced transfer of protons from CS to β-GP, neutralizing CS and allowing attractive interchain forces to form a physical gel [112]. Additionally, the ionic interaction between CS chains and β-GP, the breaking of intrachain hydrogen bonds upon heating, enhanced hydrophobic interactions among chains after neutralization, and stronger dehydration of chains by the glycerol component of β-GP all contribute to facilitating these hydrophobic interactions (Figure 5) [108,113,114].

The time required for gelation is a critical factor in the use of CS-based hydrogels, with several factors, such as the concentration of CS and other components in the hydrogel, influencing this process. Increasing the CS/β-GP ratio accelerates the gelation process [94].

Additionally, β-GP elevates the pH to the physiological range of 7.0–7.4, preventing instant precipitation or gelation and facilitating controlled gel formation upon temperature increase [94]. The study by Taherian et al. demonstrates how commercial CS with three different molecular weights (110 kD; 166,7 kD; 250 kD) and high DD (91.6–95.6%) can be neutralized closed to physiological pH using β-GP. This process produces a liquid solution at room temperature that forms a hydrogel upon subsequent heating to body temperature. The study found that the strength of the gel network is determined by the molecular weight of the CS, with higher molecular weights resulting in stronger gels. Additionally, investigations into the retention of the hydrogel in various mediums revealed that water had the greatest affinity for maintaining the integrity of the gel network [115].

However, the application of CS/β-GP thermosensitive hydrogel has been limited by challenges such as slow gelation, weak mechanical resistance, and poor cytocompatibility. To enhance the physicochemical and biological properties of these hydrogels, sodium hydrogen carbonate (NaHCO_3_) can be used in combination with β-GP [116] or phosphate buffer [109] to produce high-strength, CS-based hydrogels. Notably, Assaad et al. found that the transition from sol to gel is influenced by pH sensitivity, while the gelation time is affected by temperature variations. However, as the concentration of the gelling agent increases, the gelation kinetics become less sensitive to temperature changes [109]. Moreover, some studies have developed 3D-printed thermosensitive CS hydrogels. Recently, Rahimnejad et al. prepared a CS thermosensitive hydrogel using a mix of NaHCO_3_ and β-GP through the FRESH bioprinting approach [117].

Yun et al. developed an injectable, thermosensitive CS-based system for 5-fluorouracil (5-FU) and cisplatin (DDP) against colorectal peritoneal carcinomatosis using β-GP (10% *w*/*v*). This novel hydrogel DDS was a fluid solution at low temperatures that transitioned into a non-flowing gel upon reaching body temperature. Intraperitoneal administration of the system inhibited tumor growth and metastasis to the liver and lung in an animal model (Table 3) [118].

**Figure 6:**
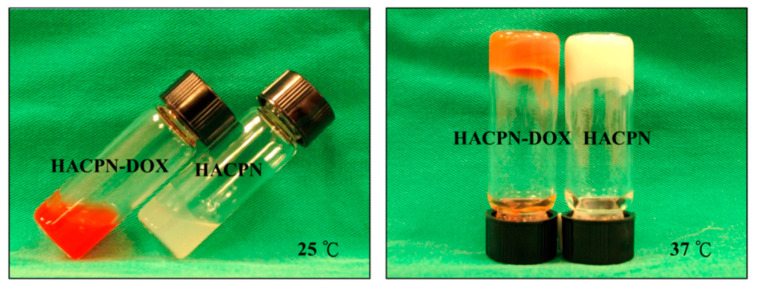
Phase transition behavior of HACPN-DOX and HACPN solutions at 25 and 37 °C. This figure is adapted from [119] with permission under CC BY 4.0 license.

Thermosensitive hydrogels are widely used in biomedical applications, often incorporating various synthetic polymers such as poly(*N*-isopropylacrylamide) (PNIPAM). The incorporation of natural polymers, such as CS, HA, or alginate, into PNIPAM can improve the biocompatibility of the thermosensitive hydrogel while retaining its thermo-responsive properties and enhancing its mechanical strength (Figure 6) [130]. The new system could improve the efficacy of intraperitoneal chemotherapy and reduce peritoneal adhesion formation [119].

### 4.2. Stimuli-Responsive Multi-Drug Chitosan-Based Hydrogels

Hydrogels have shown a potential for the delivery of orally administered drugs targeted to the colon based on pH-controlled release along the pH change in the human gastrointestinal tract [124]. The oral route of drug administration is the most preferred by patients due to its ease of use, non-invasiveness, and convenience for self-administration without the need for medical supervision. However, administering pH-sensitive drugs orally in CRC therapy presents substantial challenges. The human gastrointestinal tract is a complex system that introduces several physiological barriers to effective drug delivery [131]. The acidic environment of the stomach, with a pH typically ranging from 1 to 3, can accelerate the degradation of pH-sensitive drugs, significantly reducing their therapeutic effectiveness before they even reach the target site. Furthermore, the varying pH levels throughout different parts of the gastrointestinal tract, coupled with the presence of digestive enzymes, can further complicate the stability and absorption of these drugs [132].

To overcome these challenges, various strategies are being explored, including the use of advanced drug delivery systems such as pH-sensitive carriers. CS is well known for its pH sensitivity due to the presence of amino groups that can be protonated or deprotonated depending on the pH of the environment. However, CS-based hydrogels can lead to a burst release of the drug in the stomach due to their solubility in gastric contents. To address these limitations, pH-responsive hydrogels have been developed that release the drug after swelling at specific pH levels, thereby improving the oral bioavailability of the drug [133].

Ghobashy et al. achieved the polymerization of abundant amino groups (-NH_2_) on the backbone of CS with the carboxyl and sulfonic groups of two anionic polymers, acrylic acid (AA) and 2-acrylamido-2-methylpropane sulfonic acid (AMPS), through gamma irradiation cross-linking to obtain an amphiphilic, sterilized, and pH-sensitive hydrogel. The amphiphilic hydrogel was loaded with 5-FU, and the stimuli-responsive properties of the new system allowed for enhanced drug release at pH 7 (25.3% after 30 min; 96% after 7 h) compared to pH 1 (1.55% after 30 min) [125].

Zarbab and colleagues developed an effective biodegradable and biocompatible hydrogel (GG/PVA/CS) cross-linked with TEOS and incorporated with methotrexate (MTX), designed to be sensitive to pH variations. Their investigation of hydrogel variants with different polymer ratios (GG, CS, and PVA) and cross-linker concentrations revealed significant impacts on pore size, swelling behavior, and water-retention capacity. This hydrogel, obtained through a solution casting technique, shows potential for sustained drug release, with 96% methotrexate released within 7 h, enabling localized delivery of MTX to target colon cancer [126].

Recently, much research has focused on the synergistic effect of multiple drugs (such as two cytostatic, or the combination of chemotherapy and pain relief) in colorectal cancer therapy, as well as the development of multi-drug-loaded biomaterials [122,134].

Liang et al. developed MoS_2_ nanoflower-doped chitosan/oxidized dextran (CS/OD) hydrogels for the sequential delivery of 5-FU and methotrexate (MTX). These dual-loaded drug delivery systems release cytostatic drugs via a dual-stimuli process: near-infrared (NIR) irradiation triggers the release of 5-FU, while pH stimuli control the release of MTX. The unique, sequential delivery of these two chemotherapeutics from the hydrogels is closely related to the electrostatic attraction and the –N=CH– bonds between CS and OD. Due to the synergistic effect of the multi-drug chitosan-based hydrogels, cell viability drops to less than 10% when treated with 512 μg/mL. Moreover, cell viability further decreases to 4.9% after 30 min of NIR irradiation, which can be attributed to the effect of the synergistic, stimuli-responsive therapy [123].

Photothermal therapy (PTT) offers an alternative route for cancer treatment. This kind of therapy relies on photothermal agents with photothermal conversion capability to eliminate tumors at high temperatures, providing advantages of high precision and low toxicity [135]. However, it suffers from a lack of accumulation of photothermal agents, low photothermal conversion efficiency, and incomplete tumor elimination, leading to a very low therapeutic effect [136]. To overcome these difficulties, combined localized and dual-stimuli PTT and chemotherapy were developed. The addition of drug molecules and photothermal materials has realized multifunctional in vivo tumor PTT and chemotherapy, ensuring their in vivo biosafety [120].

Wang et al. developed an enzyme-responsive, CS-based hydrogel designed to efficiently encapsulate the hydrophobic drug imatinib (IMT) and sodium deoxycholate (a permeation enhancer). This hydrogel is intended for oral administration, where it triggers release in response to intestinal enzymes and opens epithelial tight junctions, thereby enhancing the treatment of CRC [127]. Oral administration provides an effective, non-invasive approach for the treatment of CRC.

### 4.3. Nanocomposite Hydrogels

Currently, many methods have been developed to enhance the mechanical strength and thermal stability of hydrogels, with nanocomposite hydrogels emerging as a particularly promising approach. By integrating hydrogels with nanocarriers, nanocomposites with superior properties and tailored functionality can be obtained [137]. Nanocarriers can significantly enhance drug delivery and minimize side effects, particularly for poorly water-soluble drugs. By reducing burst drug release effects and modulating drug release profiles, nanocarriers have become promising candidates for anti-cancer DDSs. However, a major challenge in vivo is the lack of colloidal stability of these carriers, leading to their rapid elimination from the bloodstream before reaching the target site [138]. To address this issue, many researchers are focusing on developing nanocomposite systems. A composite is a structural material made by combining two or more constituents on a macroscopic scale while preserving their individual properties. Nanocomposite systems based on CS can adsorb onto the surface of metal oxide NPs and stabilize their dispersion through electrostatic repulsion [139]. Additionally, this surface modification can enhance the physical, chemical, and biological properties of the NPs [140].

The co-administration of CS with drugs has been shown to enhance both transcellular and paracellular transport of drugs across the mucosal epithelium. CS, due to its positive charge, interacts directly with cell membranes, binding to them and decreasing the trans-epithelial electrical resistance of cell monolayers. This interaction increases paracellular permeability, facilitating drug transport across the epithelial barrier [141,142]. Research has demonstrated that CS solutions can increase transcellular and paracellular permeability in a reversible, dose-dependent manner. This effect is influenced by the molecular weight and degree of deacetylation of the CS, as well as by the pH of the surrounding environment [143].

In DDSs, CS-based NPs and CS-coated microspheres facilitate drug transport partly through endocytosis and transcytosis. Additionally, CS NPs have been shown to decrease transepithelial electrical resistance and increase the permeability of molecules like FITC-dextran, further indicating their impact on paracellular transport as well [144,145].

Recently, Sun et al. developed core–shell ZnO/CMC/CS bio-nanocomposite hydrogel beads using an electrostatic self-assembly method. These beads serve as a pH-sensitive carrier for 5-FU (Table 4). The ionic interactions between the carboxylate anions of carboxymethyl cellulose (CMC) and the cationic amine groups of CS enhance the hydrogel’s sensitivity to gastrointestinal conditions. Additionally, the incorporation of ZnO nanoparticles contributes to controlled drug release profiles, imparts antibacterial properties, and provides remarkable mechanical strength to the hydrogel system [146].

Nowadays, combinatorial therapy is gaining attention in tumor treatment due to its low side effects and the synergistic effect of enhanced cancer cell inhibition. Dhanavel and colleagues reported on CS/graphene nanocomposites, which are based on graphene nanosheets, as DDS. These nanocomposites are particularly effective because the large surface area and π-conjugated structure of graphene allow for the encapsulation of more than one therapeutic agent [150].

## 5. Clinical Challenges and Limitations

While CS is widely studied for its biomedical applications, including DDSs and tissue engineering, the use of CS-based hydrogels in CRC treatment remains in the early, preclinical stages. There are significant challenges to overcome in translating CS-based hydrogels into clinical practice, particularly concerning regulatory approval. Additionally, manufacturing CS-based hydrogels with consistent quality and in sufficient quantities for clinical use poses difficulties, which limits large-scale applications. The U.S. Food and Drug Administration approved CS as a GRAS (Generally Recognized as Safe) biomaterial in 2003 for use in wound hemostatic dressing due to its biocompatibility, biodegradability, and hemostatic properties. However, CS has not yet been approved for pharmaceutical use, primarily due to concerns over its source, purity, and potential immunogenicity [154,155].

In cancer therapy, ClinicalTrials.gov (accessed on 16 September 2024) reports only a few clinical trials involving CS, including studies on breast neoplasm (NCT03202446), breast cancer (NCT02967146), cancer pain (NCT02591017), prostate cancer (NCT03712371), lung cancer (NCT04218188), and advanced solid tumors (NCT03993678). Despite this, many preclinical studies have demonstrated the potential of CS-based smart hydrogels in CRC. Table 5 summarizes the current advantages and limitations of using smart CS-based hydrogels in clinical practice.

## 6. Future Perspective

CS-based hydrogels present a promising future in DDSs for CRC therapy due to their unique physicochemical and biological properties, which can be modulated through synthesis. Future research could focus on more precise therapies by functionalizing CS-based hydrogels with targeting ligands or antibodies specific to CRC cells, thereby improving efficacy and reducing off-target effects. Moreover, the development of new cross-linkers and methods for synthesizing CS-based hydrogels could enable the production of hydrogels with enhanced mechanical properties and biocompatibility.

The development of multi-stimuli-responsive smart hydrogels capable of releasing drugs in response to various triggers, such as pH changes, temperature, or enzymatic activity associated with CRC, could enable controlled and site-specific drug release, further optimizing treatment outcomes. Moreover, synergistic approaches that combine various chemotherapeutics and immunotherapeutics hold significant promise for enhancing treatment efficacy.

Advances in personalized medicine could lead to the development of customized CS-based hydrogels tailored to individual patient profiles, including specific tumor characteristics and patient-specific drug sensitivities. These hydrogels could also be utilized in 3D bioprinting to develop advanced in vitro models for anticancer drug screening and personalized medicine.

Advanced formulations may focus on reducing peritoneal adhesion formation or promoting tissue regeneration following CRC surgery, thereby improving surgical outcomes.

In summary, the future of CS-based hydrogels in CRC therapy holds significant promise. Continued research and technological advancements are expected to enhance their functionality, effectiveness, and safety, potentially revolutionizing the approach to CRC treatment and improving patient outcomes.

## Figures and Tables

**Table 3:** Chitosan-based stimuli-responsive hydrogels for colorectal cancer treatment.

Hydrogel System	Therapeutic Agent	Route of Administration	In Vitro Studies			In Vivo Studies			Ref.
			Drug Release Study	Cytotoxicity	Cell Line	Animal Model	PD	Cell Line	
CS/GP	5-FU, DDP	intraperitoneal injection	Sustained manner over an extended period, cumulative release rate of DDP is higher than that of 5-FU.	IC_50_ = 3.43 μg mL^−1^ (5-FU micelles after 48 h); IC_50_ = 6.48 μg mL^−1^ (5-FU after 48 h).	CT26	CRPC mouse model BALB/c female mice	CS hydrogel drug suppressed the growth of implanted tumor (10.33 ± 2.66, 0.49 ± 0.11 g) compared with NS group (53.83 ± 9.99, 2.31 ± 0.38 g, *p* < 0.001) and impaired tumor metastasis, as well as prolonged survival time of the tumor-bearing mice.	CT26	[118]
HACPN (hyaluronic acid-g-chitosan-g-PNIPAM)	DOX	intraperitoneal injection	At pH 7.4, 40% of the drug was released within 8 h (burst release),sustained release of DOX was observed thereafter with 80% of the drug released in 12 days.	Cell viability in the HACPN-DOX group was significantly reduced, reaching 48% and 2% of the control group after 24 and 48 h, respectively.	CT26	BALB/c mice	Suppressed tumor growth and inhibited tumor angiogenesis.	CT26	[119]
CS/MoS_2_/Bi_2_S_3_-PEG	DOX	injection	4.5% (at 37 °C and pH = 7.4), 20.3% (at 37 °C and pH = 5.4) and 16.5% (at 50 °C and pH = 5.4)	L929 cells viability treated with CS/MoS_2_/Bi_2_S_3_-PEG/DOX: 47.7% (higher compared to DOX alone 36.53%). HT29 cells viability treated with CS/MoS_2_/Bi_2_S_3_-PEG/DOX decreased to 57.12% ± 3.87% When irradiated with 1064 nm laser, CS/MoS_2_/Bi_2_S_3_-PEG/DOX, CS/MoS_2_/Bi_2_S_3_-PEG +NIR and CS/MoS_2_/Bi_2_S_3_-PEG/DOX+NIR cultured HT29 tumor cell viability declined to 55.13% ± 3.77%, 36.03 ± 3.29%, and 12.02% ± 0.41%, respectively confirming the combined tumor therapy efficiency.	HT29 L929	HT29 xenografted tumor bearing mice	The volume of the tumor was significantly reduced or even disappeared under the function of combined photothermal and chemotherapy after 14 days’ feeding.	HT29	[120]
CS/genipin/sodium salts	CUR	injection	The initial burst releases at the first 24 h were observed in all gel samples and followed by the sustained release of c.a. 1.0% to 1.8% over day 2 to 7.	Cell viability in 3T3 mouse fibroblast cell lines was above 80%, indicating high cell survival and minimal cytotoxicity under the experimental conditions.	3T3	Sprague-Dawley rat	-	-	[121]
CSDAGG	CUR, ASA	oral	A minute amount of CUR and ASA was released during the initial 2 h in the SGF (pH 1.2). In the SIF (pH 7.4), the release of ASA and CUR was 50% and 25%, respectively. In colonic fluid (pH 6.5), the cumulative release of ASA and CUR was approximately 90% and 42%, respectively, at 24 h.	At an equivalent drug concentration, the dual drug-loaded hydrogel exhibited higher cellular cytotoxicity compared to the other samples (pristine CUR, CUR-loaded hydrogel, and ASA-loaded hydrogel).	HT29	-	-		[122]
CS/OD/MTX/TFPM	5-FU, MTX	oral	Cumulative release of MTX was determined to be 10.99%, 27.53%, 21.47% and 86.51% at pH 1.2, 5.0, 6.8 and 7.4, respectively. The cumulative release of 5-FU at pH 7.4 (maximum of release of MTX) remarkably increased to 89.78% under NIR irradiation.	Cell viability < 10% after treatment with 512 μg/mL CS/OD/MTX/TFPM. The cell viability decreased to 4.9% under NIR irradiation for 30 min.	HT-29	-	-	-	[123]
CMCS/AA	5-FU	oral	at pH 1.2: 21.37–27.76%; at pH 6.5: 61.79–77.69%; at pH 7.5, 77.08–88.89% of the drug was released within 12 h	5-FU had dose-dependent cytotoxic potential and the % cell viability decreased with increasing dose per well; 5-FU retained its cytotoxic potential after loading into the hydrogel matrix; no detectable cytotoxicity on Vero cells.	HeLa, Vero cells	-	-	-	[124]
CS/AA/AMPS	5-FU	oral	release of 5-FU after 30 min at pH 1 and 7 was 1.55% and 25.3%, respectively; 96% after 7 h at pH 7.	-	-	-	-	-	[125]
GG/PVA/CS	MTX	injection	50% drug release was observed in the first 5 h and a sustained drug release of 96% in 7.25 h.	IC_50_ = 11.7 µg/mL at GG/PVA/CS +MTX concentration of 2.34 µg/200 mL	HCT-116	-	-	-	[126]
MA-CMCS	IMT	oral	After 48 h in PBS (pH 7.4), the accumulated percentage of drug release for hydrogel was 55.8%.	CS-based hydrogel was non-cytotoxic and had a good biocompatibility against normal and cancer cells; IMT-loaded hydrogel displayed a dose-dependent cytotoxicity, and cell viabilities declined with the increase of drug concentration.	LS174T, L02	Balb/c female mice	Significantly enhanced in vivo tumor inhibition (six-fold higher compared to IMT) was achieved after oral administration with IMT-loaded hydrogel.	LS174T	[127]
PAA/CSNb/bisTz-PNIPAM	5-ASA	oral	The cumulative drug release was 8.5% at pH 2.2 and reached 92% at pH 7.4 within 48 h. Additionally, the cumulative drug release from the hydrogels at 25 °C was lower compared to that at 37 °C.	cell viability exceeded 70%	HFF-1	-	-	-	[128]
ONB–CS	DOX	oral	Hydrogel exhibited higher drug release at pH 5.7 (71.75%) than at pH 7.4 (30.82%) after 24 h.	-	-	-	-	-	[129]

**Table 4:** Chitosan-based nanocomposite drug delivery systems for colorectal cancer treatment.

Nanocomposite DDSs	Therapeutic Agent	Route of Administration	In Vitro Studies			Ref.
			Drug Release Study	Cytotoxicity (If Available)	Cell Line	
core–shell ZnO/CMC/CS	5-FU		The drug accumulated release rate was <20 from ZnO/CMC/CS beads within 2 h at SGF (pH 1.2). The cumulative release reached 80% after the next 3 h at SIF (pH 6.8); at SCF (pH 7.4) for a further 3 h the release rate was still rising due to the more hydrophilic system, leading the whole state to collapse drastically.	-	-	[146]
CsDAP@ZnO	5-FU	oral	Negligible amount of 5-FU was released during the initial 2 h in SGF (pH 1.2). The release was considerably expedited from 2 to 7 h in the SIF (pH 7.4) from both the hydrogels and gradually increased in SCF (pH 6.5).	CsDAP@ZnO nanocomposite hydrogel demonstrated greater toxicity on the colon cancer cells with respect to Sap hydrogel at an equivalent concentration.	HT-29	[147]
CS/PAA/Fe_3_O_4_	5-FU	colon and rectal	At pH 7.4 in 37 °C the release rate of 5-FU from hydrogel was decreased with the increase of cross-linker and Fe_3_O_4_ NPs. Release kinetics from nanohydrogel conformed to the Weibull model.	-	-	[148]
CAR/TMC-Ag	CUR	oral	Sustained drug release reached 98.9% ± 0.9 within 24 h in pH 7.4.	High cytotoxic effect with apoptotic induction against Caco-2 cells through G2/M cell cycle arrest	Caco-2	[149]
CS/rGO	5-FU CUR	-	pH 5.0 In 72 h, 90% of the release was attained in 5-FU-loaded systems showing higher release over CUR-loaded composites.	IC_50_ = 23.8 μg/mL for dual-drug-loaded nanocomposite; IC_50_ = 37.61 μg/mL for 5-FU loaded nanocomposite, IC_50_ = 48.12 μg/mL for CUR-loaded nanocomposite. The cell viability at 40 µg/mL for the NIH 3T3 mouse embryonic fibroblast cells was found to be 80.3%.	HT-29 NIH 3T3	[150]
MCPC	CUR	oral	At pH 1.2 18% CU was released during 2h, and up to 68% release in caecal medium over 24 h.	-		[151]
CS-PLGA NPs	TA/E	intraperitoneally	-	CS-PLGA NPs significantly inhibited tumor number and tumor volume and normalized colon histology in the colon cancer.	-	[152]
CS hydrogel-coated Au NPs	PTX		-	CS hydrogel-coated Au NPs were able to increase the expression of pro-apoptotic BAX and BAD and decrease the expression of anti-apoptotic BCL2 more than PTX alone.	LS174T	[153]

**Table 5:** Advantages and limitations of using smart chitosan-based hydrogels.

CS-Based Hydrogel System	Advantages	Disadvantages
In situ gels	Localized therapy Controlled drug delivery Postoperative adjuvant chemotherapy Endoscopic mucosal resection technique for accurate removal of polyps and early-stage tumors Intraperitoneal chemotherapy Unique pharmacokinetics parameters High payload efficiency Eliminating the need for surgical removal Reduced peritoneal adhesion formation Antibacterial activity (efflux pump inhibition)	High intraperitoneal-to-plasma drug concentration ratio Low mechanical strength Slow gelation time Burst release Inability of CS to remain in solution at physiological pH Risk of obstructing the endoscopic needle during injection Large-scale production challenges Potential immunogenicity
Stimuli-responsive multi-drug hydrogels	Porous structures increase drug loading Responses to colon selectivity (pH-, enzymatic-, temperature-, redox, pressure, and mechanical stimuli) Bio-adhesiveness Enhanced drug release Antibacterial activity Targeting photodynamic and PTT therapy	Burst release in stomach when applied oral Low mechanical strength Large-scale production challenges Tendency to coagulate with protein at high pH Complex drug release control Potential immunogenicity Biodegradation rate challenges May cause localized tissue damage
Nanocomposite hydrogels	Mechanical strength Thermal stability Enhance drug delivery Minimize side effects Extend drug lifetime in the bloodstream Protection against acidic and enzymatic degradation in the gastrointestinal tract Reduce burst release Permeation enhancement Controlled drug release Improve drug-loading efficiency Stabilize NPs CS-based NPs and CS-coated microspheres facilitate drug transport partly through endocytosis and transcytosis Antibacterial activity	Complex manufacturing process Large-scale production challenges Batch-to-batch variability Rapid biodegradation Potential immunogenicity

## Data Availability

No new data were created or analyzed in this study. Data sharing is not applicable to this article.

## Abbreviations

5-ASA	5-aminosalicylic acid
5-FU	5-fluorouracil
AA	Acrylic acid
AMPS	2-acrylamido-2-methylpropane sulfonic acid
ASA	Acetylsalicylic acid
CMC	Carboxymethyl cellulose
CMCS	Carboxymethyl chitosan
CRC	Colorectal cancer
CS	Chitosan
CSDAGG	Chitosan-dialdehyde guar gum hydrogels
CUR	Curcumin
DD	Degree of deacetylation
DDP	Cisplatin
DDSs	Drug delivery systems
DOX	Doxorubicin
E	Vitamin E
FTIR	Fourier Transform Infrared Spectroscopy
GG	Guar gum
GRAS	Generally Recognized as Safe
HA	Hyaluronic acid
HACPN	Poly(*N*-isopropylacrylamide)-based hydrogel
IMT	Imatinib
MTX	Methotrexate
NMR	Nuclear Magnetic Resonance
NPs	Nanoparticles
OD	Oxidized dextran
ONB	*Ortho*-nitro benzyl
PAA	Poly (acrylic acid)
PEG	Poly(ethylene glycol)
PNIPAM	Poly *N*-isopropyl acrylamide
PTT	Photothermal therapy
PVA	Poly(vinyl alcohol)
SCF	Simulated colonic fluid
SGF	Simulated gastric fluid
SIF	Simulated intestinal fluid
TA	Tannic acid
TJs	Tight junctions
Tzs	Tetrazines
β-GP	β-glycerophosphate
